# Supplementary material for: Effects of bouncing the barbell in bench press on throwing velocity and strength among handball players
Source: PLoS One. 2021 Nov 19;16(11):e0260297. doi: 10.1371/journal.pone.0260297 (PMC8604300; doi:10.1371/journal.pone.0260297)
Supplement: S1 Table — (PDF) [file pone.0260297.s001.pdf]

FP.nr	Gruppe	Pre_MB20	Pre_MB30	Pre_MB40	Pre_MB50
1	1 (bounce)	270,5	262,2	254,1	
3	1	355,2	405,9	451,2	415,2
5	1	384,9	494	540,7	545,5
8	1	307,3	380,7	419,3	390,7
9	1	271,7	321,7	313,6	277
11	1	294,5	344,8	392,1	359,3
14	1	376,9	489	498,9	462,5
2	2 (uten bounc	300,2	333,9	321,4	266,7
4	2	388,9	465,8	524,9	524,1
6	2	259,9	284,2	287,7	229,8
7	2	253,3	291	290,8	209,7
10	2	275,1	337	334,6	296,1
12	2	190,4	180	72,3	
13	2	340,6	434,5	446,5	434,1
**15**	**2**				

Pre_MB60	Pre_UB20	Pre_UB30	Pre_UB40	Pre_UB50	Pre_UB60	Post_MB20
	231,7	249,1	197,1			198,8
305,2	312,3	388,2	404,2	341,8	272	391,6
539,2	390,9	455,5	482,6	474,7	426,9	429,6
339,8	306,2	365,8	382,9	348,2	279,6	314,4
	276,9	309,5	302,7	238,9		283,2
346,6	265,7	331,7	332,2	333,9	277,8	306
397,4	364,3	432,4	410,7	399,8	328,4	369,9

	292,8	320	323	284,5		343,2
494,4	388,9	459,5	475,8	478,2	446,8	405,3
140,3	259,2	279,1	277,9	238	165,3	266
	252,6	293	273,1	129,8		279,3
205,6	264,9	314,5	315,5	273	171,2	283,7
	184,3	176,9	91,9			285,6
375,8	319,3	364,3	391,6	392,6	316,1	381,4

Post_MB30	Post_MB40	Post_MB50	Post_MB60	Post_UB20	Post_UB30	Post_UB40
193,3	121,4			190,6	174	90,6
474,2	518,2	451,3	352,9	351,5	435,6	431,1
548,6	591,3	660,2	685,7	431,1	509,2	545,1
396,8	427,7	407,9	348,7	315,9	369,8	402,6
309,6	332,3	265		280,7	315,3	298,8
362,5	391,8	412,7	342	281,7	343,2	356
449	474	460,2	387,7	362,4	409,3	429,2

383,8	381,8	272,8		327,5	372,6	370,5
486,6	558,9	570,1	542	387,5	464,2	501,5
286	285,1	263,5	105,1	267,3	306,7	305,9
333,9	296,8	238,1		279,7	303,5	290,1
339,9	337,6	304,1	299,7	287,5	330,3	340,5
284,1	254,2	168,9		234,1	276,3	251,7
478,1	498,7	487,5	478,9	357,3	434,2	454,7

Post_UB50	Post_UB60	Pre-1steg	Pre-3steg	Post-1steg	Post-3steg	Pre-1RM
		76,67	84,78	78,90	85,90	37,5
429,5	352,9	85,80	91,53	85,47	94,07	70
552,2	573,7	87,90	97,47	91,03	98,60	90
367,5	321,9	85,57	97,60	89,97	97,33	77,5
255,7		84,17	90,67	90,67	89,87	60
343	309,5	84,50	88,33	90,47	96,60	82,5
436,7	324,2	88,27	98,87	96,50	102,07	70
		**84,70**	**92,75**	**89,00**	**94,92**	**69,64**
286,4		77,07	81,83	78,97	82,70	60
502,8	466,1	93,93	105,47	97,60	105,60	90
263,6	179,9	77,27	82,87	83,37	85,80	65
237,4		71,53	77,50	76,40	84,90	57,5
335,6	263,9	78,00	87,77	82,23	88,40	65
191,3		85,33	92,03	90,23	97,53	50
455,5	419,5	84,93	90,23	88,63	96,17	75
		**81,15**	**88,24**	**85,35**	**91,59**	**66,07**

Post-1RM	Height	body mass
42,5	173	60,9
80	174	62,6
105	182	86,0
82,5	191	80,0
60	179	66,0
85	172	71,0
75	173	69,0
**75,71**		**70,79**
67,5	180	70,0
97,5	192	92,0
70	174	62,6
60	179	69,3
75	182	73,0
55	174	64,0
85	192	82,0
**72,86**	**187**	**70,0**
