# Supplementary material for: Effects of bouncing the barbell in bench press on throwing velocity and strength among handball players
Source: PLoS One. 2021 Nov 19;16(11):e0260297. doi: 10.1371/journal.pone.0260297 (PMC8604300; doi:10.1371/journal.pone.0260297)
Supplement: Attachment — Submitted filename: Response to reviewers.docx [file pone.0260297.s002.docx]

We would like to thank the reviewers for your valuable comments and suggestions. We believe they have contributed to improve the quality of the manuscript. Under you will find our point-to-point answers and we have made changes in the manuscript accordingly.

Reviewer #1: Abstract
Line 21: Remove the bracket.

On behalf of all authors, we apologize for this typo. The bracket has been removed.

Introduction
Line 77-78: I would suggest adding the term stretch-shortening cycle to this sentence such as for example: “A drop-jump may result in high level of force prior to the ascending phase, WITH A STRETCH-SHORTENING CYCLE TYPE ACTION THAT STORES the elastic energy, …”
Solid clarification of our point. The sentence has been re-written accordingly. Thank you.

Methods
Participants
Since you were using a sample of convenience (two handball teams), then you probably did not calculate a statistical power analysis to determine the appropriate number of subjects to achieve sufficient statistical power. Please calculate a post-test statistical power analysis so the reader knows the extent of statistical power of this study’s measures.

Based on the 7m throwing velocity, we have calculated the statistical power and the sample size needed to detect significant difference between the groups. This has been added in the discussion (ln. 365 – 368) to elaborate on our speculations and limitations of the present study.

Briefly, the statistical power was 44% and a minimum of 12 subjects was required in each training group.

Line 119-120: You might want to remind the readers that the handball players were experienced with resistance training and were familiar with their 1RM and thus the self-reported 1RM should have been accurate.
Good point. The sentence “Of note, the handball players were experienced with resistance training and were tested in 1-RM frequently (e.g., 3 – 5 times) each year” has been added to the manuscript.

Line 125-131: How did you determine or ensure a similar bounce. Although you mention lowering the bar in a controlled manner, it is possible that some individuals would incorporate a far greater bounce than others. Would this not insert a high degree of variability into the training routine?

It`s good question and difficult to provide an 100% accurate answer as we did not include measurement of the vertical descending displacement for each subject. Importantly, the instructions were “the goal is to generate as high velocity during the ascending phase as possible, with a fast but controlled lowering velocity”. Furthermore, we were more interested in ecological perspective and the “proof of concept”. That being said, we cannot reject the hypotheses of individual differences which has been added as a possible explanation in the discussion (ln 280 -284).

By “control” we referred to “not dropping the barbell from the starting position”. One familiarization session was conducted before the pre-test to ensure proper bouncing. Furthermore, one test leader attended as many training sessions as possible given the Covid-19 regulations to make sure a proper bounce was conducted. That being said, we cannot reject your hypotheses that some subjects (greater chest volume, less fear, and greater arching) may generated a far greater bounce.

In the pilot test using the same instructions and loads, we measured a 3.6 - 4.7cm longer vertical displacement in the descending phase. Typically, greater loads resulted in less bounce (probably as result of pain and fear). Furthermore, the training load was 40 – 60% of 1-RM (See table 2). We therefore hypothesized that the loading would result in different amount of bounce, but that during the training intervention, the subjects would conduct more similar bouncing across the loads.

In the pilot testing, we also included a metronome to control the lowering speed, but experienced that the subjects` attention was towards the metronome and not to conduct a bounce or a maximal explosive bench press throw.

Of note, our research group are examining your hypotheses, but not on an individual level but the descending instruction. Using the peak power loads, the subjects (resistance trained men) are being asked to lower the barbell slow, medium, and as fast as possible with and without the bounce. We passed 20 subjects yesterday and are aiming to reach 30. That being said, from these data we could calculate individual variation of the amount of bounce using the three lower velocities.

In the manuscript, we have added some sentence in the methods (ln 170-171) and discussion (ln 280-284). Thanks.

Line 138-142: How was power monitored? Was there a video analysis to monitor bar velocity or was there a line attached from the barbell to a device to monitor velocity? Table 3 illustrates the Watts but I do not see or must have missed where you explained how watts were calculated.
We apologize for this mistake and acknowledge your observant reading skills. We used a linear encoder attached to the barbell to measure the average velocity (e.g., from the start of the ascending phase to barbell`s highest position). Please see ln. 143 – 148 for further details.

Line 162: unsupervised is one word, no hyphen necessary.
Thank you, changed accordingly.

Line 281-284: I would add an explanation to this information that the individuals in that study may not have had the relative lower body strength to rapidly absorb or stretch the musculotendinous unit and then transition to a propulsive contraction. This would be in alignment with your previous statement that your subjects may not have been strong enough. In addition, it could be speculated that if your subjects had not trained specifically for eccentric strength then perhaps there might have been a specific eccentric strength deficit (i.e., training specificity). With a lack of prior eccentric training, individuals can have a lack of balance between concentric and eccentric strength.

Added, as suggested (ln. 297 – 306).

Line 336: I have never seen this word before: contrastive; and I have been speaking only English for 64 years. Please change to “In contrast,…”.

Google translate has… 😊. Changed to “In contrast, … and later the sentence was deleted….

Line 331-350: The power output differences were not significant as reported in the results (no interaction (F = 0.066 – 2.477, p = 0.142 – 0.802) or main effect for group (F = 0.453 – 1.467, p = 0.254 – 0.513)). This paragraph makes the reader think that something significant did happen. Without significance then the variability was greater than the mean difference and thus essentially there is no real difference. For example, the authors state: “As power is the product of both force and velocity, a possible explanation of the findings could be that the BPT emphasized the subjects’ ability to generate force more than the BPTbounce.” But if there is no significance then there are no sig different findings and this there is no need for an explanation of these non-significant findings. Please remove this paragraph or change the rationale and message of the paragraph to reflect the lack of significant difference.

Thank you for bringing this to our attention. Yes, there were no interactions or main effects for group, but a main effect for time for some of the loads. Importantly, post hoc tests have only been conducted within a group (pre – post) and not between groups. However, your example “as power is the product of…” we acknowledge that the paragraph needs to be re-written and clarified. Therefore, we have re-written the paragraph, removed major parts of it, and clarified the findings using words as “no significant difference”. Please ln 351 – 362.

Thank you for taking the time and sharing your knowledge in this review proses. Your comments and suggestions will be included and kept in mind in future papers. Thank you!

Reviewer #2: This is a well written, reasoned, conducted and analysed study. The hypotheses are clear, the findings are as well, the interpretation is sound and based on the results and appropriate literature, there is no interpretative over-reach, and the limitations are stated. This is a tight study that adds to the body of literature on sport specific power training and i have only minor comments below. Well done.

Thank you so much!

Abstract
Instead of stating “no difference” please state no significant difference if it can fit into the abstract word limit.

We managed to add “no significant difference” in abstract. Good point, added as suggested.

Introduction
Line 67-68: please change “creates a momentum to help accelerating” to “creates momentum to help accelerate”

Changed, thank you

Procedures
Line 125 please change “barbell in the chest.” to “barbell off the chest.”

Changed, thank you

Results
In all cases where you state “no difference” or “no interaction” or similar please change to “no significant difference” or “no significant interaction”

Added, as suggested.

Discussion
Line 291 please change “no increase” to “no significant increase”

Added, as suggested.

Thank you for taking the time and sharing your knowledge in this review proses. Your comments and suggestions will be included and kept in mind in future papers. Thank you!
